# Synergistic Anti-Ageing through Senescent Cells Specific Reprogramming

**DOI:** 10.3390/cells11050830

**Published:** 2022-02-28

**Authors:** Rui Chen, Thomas Skutella

**Affiliations:** Group for Regeneration and Reprogramming, Medical Faculty, Department of Neuroanatomy, Institute for Anatomy and Cell Biology, Heidelberg University, 69120 Heidelberg, Germany; rui.chen@uni-heidelberg.de

**Keywords:** ageing, senescence, senolytics/senostatics, p16^Ink4a^, p19^Arf^, p21^Waf1/Cip1^, SASP

## Abstract

In this review, we seek a novel strategy for establishing a rejuvenating microenvironment through senescent cells specific reprogramming. We suggest that partial reprogramming can produce a secretory phenotype that facilitates cellular rejuvenation. This strategy is desired for specific partial reprogramming under control to avoid tumour risk and organ failure due to loss of cellular identity. It also alleviates the chronic inflammatory state associated with ageing and secondary senescence in adjacent cells by improving the senescence-associated secretory phenotype. This manuscript also hopes to explore whether intervening in cellular senescence can improve ageing and promote damage repair, in general, to increase people’s healthy lifespan and reduce frailty. Feasible and safe clinical translational protocols are critical in rejuvenation by controlled reprogramming advances. This review discusses the limitations and controversies of these advances’ application (while organizing the manuscript according to potential clinical translation schemes) to explore directions and hypotheses that have translational value for subsequent research.

## 1. Introduction

Ageing can be defined as a time-dependent decline in the functionality of the body. At the cellular level, its essence can be seen as a gradual loss of normal cell function accompanied by a series of ageing phenotypes [1,2]. Stress factors such as telomere dysfunction, DNA damage, oncogene activation, and organelle dysfunction accelerate the progression of senescence at the cellular level, spreading through the cellular microenvironment and accelerating organ dysfunction throughout the tissues, culminating in the loss of all vital functions of the body (organ failure).

Interestingly, there is a self-balancing of repair after injury and renewal after ageing in the organism itself. These balancing mechanisms include the conversion of resting stem cells to progenitor cells in various tissues within dynamic homeostasis. Alternative or anti-dysfunctional mechanisms act at the organelle level of dysfunction (e.g., alternative mitochondrial glycolytic and glutamine metabolic pathways, activation of nuclear repair through nuclear damage) [3,4,5]. At the tissue level, the senescence-associated secretory phenotype (SASP) has some anti-ageing potential in addition to being a senescence-promoting factor (e.g., interleukin-6 (IL-6) can enhance tissue repair by promoting reprogramming [6,7]; interleukin-1 (IL-1) can promote the clearance of senescent cells by immunopurified NK cells [8,9,10]). It is important to note that there is no evidence that anti-ageing can be achieved by exploiting the beneficial aspects of ageing itself. This review aims to show that reprogramming interventions on senescent cells have the potential to retain these valuable components compared with senescent cell removal. There is also the possibility that reprogramming can be self-modified by the above result, in which senescence promotes reprogramming: the same expression level of reprogramming factors may be more effective in the senescent environment. In contrast, the efficiency of reprogramming is reduced when the senescent environment is mitigated or reversed, thus avoiding the harm caused by over-induction.

To explore the anti-ageing potential of this balance, this review will discuss the possibility and feasibility of attenuating senescence through intercellular interactions as follows:

- To propose synergistic anti-ageing in order to clarify the idea of combined anti-ageing with multiple rejuvenating factors and to serve the next research on new key pathways that can be applied in combination with known anti-ageing pathways.
- The hypothesis of a youthful secretory phenotype is proposed to generalise the anti-ageing factors (particularly, NAD^+^, eNAMPT, GSTM2, etc. are involved in whole body anti-ageing by regulating the circulating NAD^+^/NADH balance) found in the secretome of young blood and young cells and to serve for future clinical translation and co-application.
- To propose the hypothesis that controlled reprogramming (defined as the induction of Yamanaka factors expression to reverse the ageing phenotype of cells but without iPSCs-induced pluripotent stem cell formation) may synergistically anti-age by a youthful secretory phenotype.

## 2. The Characteristics of Ageing and their Potential for Translation

### 2.1. “Asynchronous Effect” in Ageing

As we age, the ageing of different organs and tissues is not synchronized, and the parenchymal cells that perform the biological functions of organs/tissues are in different stages of senescence [1].

It has been shown in mice that plasma cells and the antibodies they secrete infiltrate various organs, appearing in the kidney, heart, liver, muscle, fat, lung, and thymus [2]. This implies that ageing in one organ may trigger or accelerate ageing-related chronic inflammation and dysfunction throughout the body via the systemic circulation. Furthermore, when mice reach middle age, immune cells (T and B cells [2], M1 macrophages [11,12]) are extensively activated in adipose tissue [2]. These studies suggest that ageing and immunity are inextricably linked, and ageing is an “asynchronous” process. On the other hand, adipose tissue is one of the first areas of the body to show senescence-related phenotypes (inflammatory cell infiltration and the appearance of senescence-related secretory phenotypes) [1,2].

In fact, the removal of senescent cells can reduce the adverse effects of pre-senescent fractions (e.g., senescent cell ablation-senolytics) [13]. As cellular senescence is not synchronized, the senescence microenvironments originating from pre-senescent cells can cause a vicious cycle (the senescence of one organ promoting the decline throughout the body) at the tissue level [1,2]. The “old factors” that promote ageing may be diluted or suppressed by another “rejuvenating factor (existing in young blood as well as being secreted by a heterogeneous subgroup of ageing cells)”, thus acting as a rejuvenating agent [14,15]. Through transplanting pre-senescent adipocytes, it was found that the senescence of a small number of adipose precursor cells was sufficient to induce organ senescence in juvenile mice. The removal of transplanted senescent cells from young mice and naturally senescent cells from naturally senescent mice by intermittent oral senolytics improved ageing (organ function enhanced, survival increased by 36%, and the risk of death reduced by 65%) [13].

The heterogeneity of ageing is reflected on the one hand in the fact that chronic inflammation differs in different tissues, specifically in the fact that SASP has different levels in different tissues [2]. On the other hand, it is reflected in the different sequences of appearance of senescent cells and the different rates of accumulation [1].

For example, in the kidney, where senescent cells increase significantly with age, Cu/Zn-superoxide dismutase (Sod1) knockout mice result in high levels of oxidative cellular senescence [16]. Senescence-related secretory phenotypes (particularly IL-6 and IL-1β) are also significantly increased [16]. The higher levels of circulating cytokines suggest that the accelerated senescence phenotype may be due to increased inflammation caused by the accelerated accumulation of senescent cells [17]. The accumulation of senescent cells, in turn, caused an increase in chronic inflammation [16].

Remarkably, asynchrony of senescence persists even in the same ageing cells (fibroblasts in aged mice) [14]. Different subpopulations with different secretory phenotypes influence the rate of wound healing in vivo by affecting reprogramming efficiency [14].

Taken together, it is feasible to intervene in pre-senescent tissues for overall benefit by taking advantage of the asynchrony in ageing [18]. It is also known that the microenvironment has a key influence on the state of cell ageing (cells exposed to the secretome of senescent cells will age faster) [13]. Rather, improving the cellular micro-environment (increasing the secretory phenotype of young cells) to combat ageing is a promising direction [19]. In particular, adipose tissue is one of the first to be affected by ageing (also having a key influence on the inflammatory state associated with ageing) [1,2].

### 2.2. “Synergistic Effect” in Anti-Ageing

It is shown that blood therapy involves several different anti-ageing factors (GDF11 [20], GPLD1 [21], clusterin [22], Klotho [23], etc.). On the one hand, it leads to controversy [19], but on the other hand, it also suggests that greater benefits may be achieved by synchronizing multiple factors to combat ageing. Synergistic anti-ageing is a phenomenon in which the combined modulation of multiple anti-ageing factors produces a higher effect than the sum of the effects of modulating any of them individually. Therefore, studies of ageing patterns are instructive when discovering critical pathway of anti-ageing with synergistic potential (e.g., in simple eukaryotes).

Some yeast cells show significant nuclear stability changes during cell ageing and exhibit ribosomal senescence, while others develop mitochondrial dysfunction. In yeast with a ribosomal senescence pattern, overexpression of Sir2 (a lysine deacetylase that contributes to ribosomal DNA silencing) would extend the average lifespan of the yeast [24,25]. Overexpression of Sir2 and Hap4 prolongs lifespan by producing synergistic rather than additive effects [26]. A similar synergistic effect is observed when the fob1Δ longevity mutant enhances rDNA stability combined with Hap4 overexpression [25]. This model also explains the anti-ageing synergy between caloric restriction, promoting heme activator protein (HAP) and Sir2 [26]. These two seemingly independent lifespan factors can be understood as two key anti-ageing nodes and targets with synergistic anti-ageing effects. Both could be considered critical anti-ageing nodes that need to be regulated simultaneously.

In another study using *Caenorhabditis elegans* (*C. elegans*) as a model, an essential regulator gene called CYC-2.1 (a nematode cytochrome C ortholog), a cytochrome strongly associated with mitochondrial ageing was identified. Reducing CYC-2.1 expression activated the “unfolded protein response” in mitochondria, promoting their division and thus significantly extending nematode lifespan [27]. The rsks-1 (the *C. elegans* ribosomal S6K ortholog) mutation increased average lifespan by 20%, the daf-2 (a nematode insulin growth factor 1 receptor ortholog) mutation increased the average lifespan by 169%, and the daf-2 and rsks-1 double mutation increased the average lifespan by 454% over the wild type; thus, the increased longevity of the daf-2 and risk-1 double mutants is not simply additive but has a synergistic effect on longevity [28]. On the other hand, TOR (target of rapamycin) regulates mRNA translation levels via ribosomal S6 kinase (S6K) [29]; therefore, it demonstrates a significant synergistic anti-mitochondrial ageing effect of the IIS (insulin/insulin-like signalling) and TOR [27]. This study also suggests that synergistic regulation of ribosomal protein genes and mitochondrial function could increase the synergistic effects of key anti-ageing factors to a greater extent. It also implies that mitochondrial function regulation could be achieved through metabolic reprogramming induced by remote intercellular regulation and could elicit a broader response from various immune-metabolic cells and organs via an anti-ageing secretory phenotype.

Reactive oxygen species (ROS)-induced DNA damage response (DDR) activates mTORC1 through direct phosphorylation of protein kinase B (PKB/Akt) by ATM, and activated Akt directly phosphorylates the TSC1/TSC2 complex, in this way activating mTORC1. Activation of mTORC1 promotes ROS-dependent DDR, and, through the mitochondrial biogenesis transcriptional co-activator peroxisome proliferator-activated receptor-gamma coactivator-1beta (PGC-1β), promotes ageing phenotypes (e.g., ASAP), ultimately resulting in ROS-mediated DDR activation (upregulation of DDR protein γH2A.X) and cell cycle arrest (with reduced expression of p21^Waf1/Cip1^ and p16^INK4a^) [4]. This apparent vicious circle, if not broken, results in increasing senescence. Thus, improvements in mitochondrial function and altered redox status are key factors in breaking this impasse.

Mitochondrial dysfunction-associated senescence (MiDAS) leads to a reduction in the NAD^+^/NADH ratio, which leads to activation of AMPK and p53, which leads to both growth arrest of senescent cells (caused by p53 activation, with pyruvate preventing MiDAS growth arrest but restoring NF-κB activity) and AMPK-mediated p53 activation reducing IL-1 secretion [3]. This implies that there are multiple and intricate patterns of ASAP such that reprogramming strategies dependent on IL-6 boosting efficiency can break the mitochondrial dysfunction (MiD)-ROS-dependent DDR vicious cycle by modulating the NAD^+^/NADH ratio (possibly in parallel with the pyruvate response) while responding to the promotion of the senescence microenvironment. It implies that the emergence and persistence of rejuvenation microenvironments in the blood [30] (e.g., endocrine rejuvenation microenvironment), as well as immune rejuvenation microenvironments, are crucial because of their systemic nature (affecting almost all cells) and the breadth of their effects (participating in nearly all rejuvenation-related pathways).

**Figure 1:**
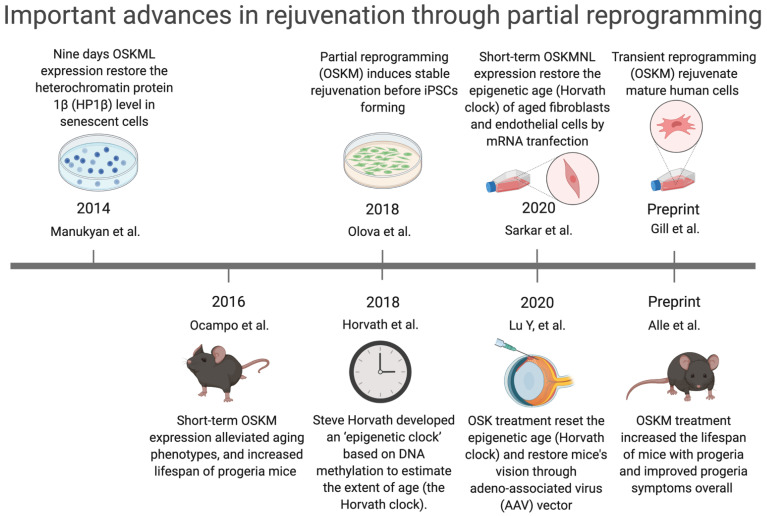
Important advances in rejuvenation through partial reprogramming. Manukyan et al.: Nine days OSKML expression restored the heterochromatin protein 1β (HP1β) level in senescent human fibroblasts [32]. Ocampo et al.: Short-term OSKM expression alleviated ageing phenotypes and increased lifespan of the progeria mice (LAKI 4F mice) [31]. Olova et al.: Partial reprogramming (OSKM) induced stable rejuvenation of adult human fibroblasts before iPSCs forming [34]. Horvath et al.: Steve Horvath developed an “epigenetic clock” based on DNA methylation to estimate the extent of age (the Horvath clock) [35]. Sarkar et al.: Short-term OSKMNL expression restored the epigenetic age (Horvath clock) of aged human fibroblasts and endothelial cells by mRNA transfection [36]. Lu Y et al.: OSK treatment reset the epigenetic age (Horvath clock) and restored mice’s vision through adeno-associated virus (AAV) vector [37]. Gill et al.: Transient reprogramming (OSKM) rejuvenated mature human cells [38]. Alle et al.: OSKM treatment increased the lifespan and improved premature phenotypes in the progeria mice [39].

Short-term exposure to Oct4, Sox2, Klf4, and c-Myc (OSKM) (also called “Yamanaka factors”) reverses the ageing phenotype of cells [31], demonstrating that senescence is reversible [32]. This means that rejuvenating senescent cells is a new strategy for disrupting the vicious cycle of ageing by creating dynamic rejuvenation homeostasis in multiple pathways together. However, it is important to note that premature termination of reprogramming can lead to failure in the rejuvenation of MSCs [33]. Thus, partial reprogramming (defined as inducing Yamanaka factors expression to reverse the ageing phenotype of cells but without iPSCs-induced pluripotent stem cells forming) is a potential anti-ageing intervention [31] (Figure 1).

## 3. Strategies for Reversing Senescence and the Potential Underlying Mechanisms

### 3.1. Reprogramming-Based Therapies to Reverse Senescence

Partial reprogramming simultaneously lengthens telomeres, inhibits p53, and restores mitochondrial function [31]. Interestingly, the telomerase reverse transcriptase overexpression in transgenic mice (Sp53/Sp16/SArf/Tg Tert mice) showed improved tumour resistance and was found to prevent ageing-related degeneration (mainly atrophy) and inflammatory processes, higher blood levels of IGF1, and a reduction in γ-H2AX foci. Increased glucose tolerance and neuromuscular coordination cause a longer average lifespan [40]. The telomere–p53–PGC pathway and its downstream gene network regulate the functional state of multiple organs and ageing: increased levels of p53 (Trp53) lead to inhibition of peroxisome proliferator-activated receptor-gamma coactivator-1 alpha (PGC-1α). The germline deletion of p53 fully restores PGC network expression; PGC-1α expression restores mitochondrial respiration, cardiac function, and glucose allosterism [41]. Furthermore, reducing peroxisome proliferator-activated receptor-gamma coactivator-1beta (PGC-1b) attenuates cellular senescence-related phenotypes [4]. This implies that short-term cyclic expression of OSKM can rejuvenate senescent cells’ epigenome in vivo, reduce p16^Ink4a^ and SASP, and affect various senescence-related regulatory pathways (such as mitochondria dysfunction, DNA damage, impaired protein folding, telomere shortening, and inflammation [31]), thus exerting a synergistic anti-ageing effect.

**Figure 2:**
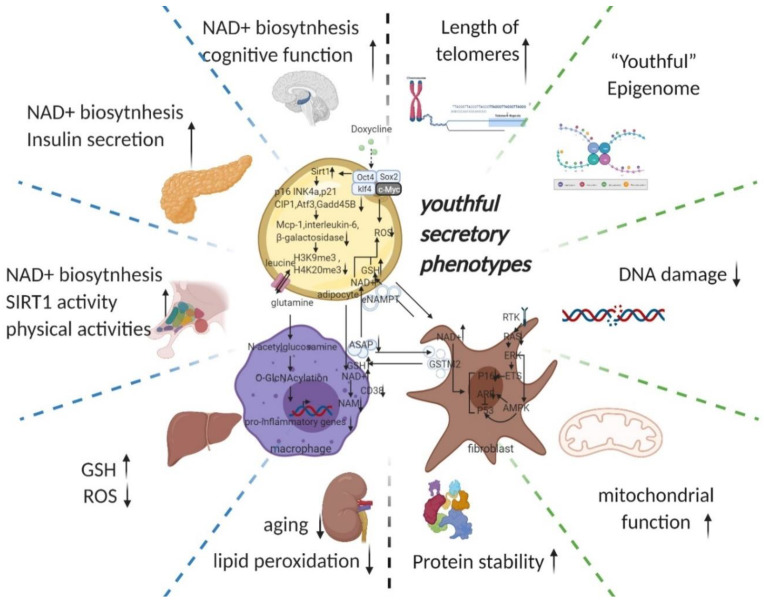
Potential intercellular mechanisms related to senescent cells specific reprogramming. Senescence in adipose precursor cells can be improved directly or indirectly (via reduced p21 and p16 pathways by overexpression of Sirt1 [43,55]) by doxycycline-induced overexpression of OSKM. The reversal of senescence by reprogramming can comprehensively improve senescence indicators (decrease in p16, p21, senescence-associated β-galactosidase, etc.) and can, at the same time, ameliorate senescence-associated secretory phenotypes (decreased Mcp-1 and Il-6, MMP13) and even improve histone methylation status (decrease in H3K9me3, H4K20me3) [31]. With the rejuvenation of adipose tissue (telomere lengthening, phenotypic rejuvenation remodelling, and promotion of gene damage repair), the upregulation of adipocyte glutaminase 1 [56] is reversed and the tissue is therefore rescued from the glutamine depleted state caused by ageing. Increased levels of glutamine will improve the chronic inflammatory state associated with ageing on a systemic scale by reducing the transcription of pro-inflammatory genes in macrophages in adipose tissue [48]. This means that the production of senescence-associated secretory phenotypes is reduced, thereby favouring the maintenance of a youthful state in surrounding fibroblasts, adipocytes, and themselves. The reprogramming also promotes the production of secretory eNAMPT in extracellular vesicles. By altering the NAD+ content of cells to regulate their mitochondrial metabolic state and redox homeostasis, eNAMPT promotes the rejuvenation of various cells throughout the body (improves pancreatic and hypothalamic secretion phenotypes, thereby amplifying anti-ageing effects via hormones) [49,57]. Macrophage rejuvenation not only improves the rejuvenation of the systemic secretory phenotype but also attenuates NAD^+^ degradation through reduced CD38 expression [11]. This may have a synergistic anti-senescence effect with eNAMPT. NAD^+^ and a rejuvenated secretory phenotype (possibly through metabolic reprogramming or cell rejuvenation via ERK–AMPK regulation of P16 and P53) improve the GST secretory capacity of fibroblasts. Delivery of GST to organs throughout the body via extracellular vesicles improved cellular redox homeostasis, resulting in a promising anti-ageing effect (improves liver redox status and kidney ageing) [51]. Taken together, local reprogramming through systemic cellular communication (eNAMPT, YSAP, and GST, etc.) produces synergistic anti-ageing effects (improvement in redox and metabolic imbalances caused by mitochondrial senescence and protein instability caused by ribosomal senescence). However, it is worth noting that further studies are needed to determine whether reprogramming can produce sufficient alterations in the secretory phenotype and whether intercellular communication can alter the secretory phenotype of adjacent cells. (black arrow: direct stimulatory, round arrow: cycle, dotted arrow: tentative stimulatory, down faded arrow: decrease, up faded arrow: increase; the grey dotted lines depict macro-level improvements on the left and micro-level improvements on the right, both separated by green dotted lines).

Due to the “asynchronous” character of ageing, senescent cells reprogramming preferentially affects the tissues that are first influenced by ageing (e.g., adipose tissue, the immune system, and fibroblasts [1,2]). We, therefore, start our discussion with adipose tissue (Figure 2). Ageing is often accompanied by a decline in subcutaneous adipocytes marked by the depletion of adipose precursor cells [42], which in turn causes a change in fat tissue distribution—i.e., more visceral white fat and less brown fat [43,44] as well as ectopic fat deposits [45]. This transformation leads to a vicious circle of producing an ageing microenvironment through an imbalance in the inflammatory state and cellular metabolic state associated with ageing and, consequently, a disruption of cellular homeostasis (proteostasis) [46].

Senescence of adipose precursor cells (caused by sirtuin 1 reduction) leads to the accumulation of senescent adipocytes [43], which secrete pro-inflammatory factors that constitute the first part of the senescent microenvironment and cause chronic inflammatory infiltration of adipose tissue [47]. As ageing redistributes fat (visceral fat increases), senescent adipose tissue carries the chronic inflammatory state associated with senescence (Mcp-1 and Il-6) throughout the body and gradually accumulates.

Increased white adipose tissue causes a decrease in glutamine levels in adipose tissue, leading to increased macrophage glycolysis in adipose tissue, increased pro-inflammatory transcription, and secretion of large amounts of SASP into the peripheral microcirculation, generating a second part of the senescent microenvironment [48].

M1 macrophages in senescent white adipose tissue consume large amounts of NAD+ [11,12], and adipocytes secrete less eNAMPT due to senescence [49,50], resulting in a systemic NAD^+^/NADH ratio imbalance (lower), which accelerates mitochondrial dysfunction-related senescence in cells throughout the body [3], resulting in an imbalance in energy metabolic status (glycolysis increase) and creating the third part of the senescent microenvironment.

Mitochondrial metabolic disorders cause enhanced glycolytic pathways and cellular redox disorders, resulting in systemic redox disorders [3,4]. Systemic fibroblasts under the influence of the first three parts of senescence and their own senescence, decrease GST secretion, exacerbating systemic peroxidation and creating the fourth part of the senescence microenvironment [51].

The redox disorder strongly affects genomic stability [5], generating many misconfigured proteins, which form aggregates that are expelled from the cells and adipocytes, which also discharge aged mitochondria, forming the fifth part of the senescent microenvironment [52,53,54].

Protein mismatches reach the upper limit of cellular discharge and continue to accumulate, damaging the cell’s genetic repair mechanism, which in turn disrupts all cellular functions into a state of irreversible death, releasing waste after its death and thus creating the sixth part of the senescent microenvironment. (Therefore, the simple removal of senescent cells might not avoid the senescence signal release process during senescent cell death).

### 3.2. Potential Key Mechanisms Related to Reprogramming-Based Therapies

#### 3.2.1. Cyclin-Dependent Kinase Inhibitors (p16^INK4A^)

Multiple lines of evidence suggest p16^INK4A^ extensive involvement in the ageing process, which could serve as an alternative regulatory centre in anti-ageing strategies. Mice with low levels of cell cycle checkpoint kinase BubR1 expression suffer from an acceleration in ageing as well as high levels of p16^INK4A^ in tissues with age-related histopathology [58]. Targeted mutation of p16^INK4A^ caused an ageing delay in the BubR1 mice. This delay went on together with reduced levels of senescent cells. This reveals a connection between biological ageing and cellular senescence [59]. In INK-ATTAC mice, where senescent cells expressing p16^INK4A^ are specifically killed, the loss of senescent cells increases the lifespan and health span [60]. It has been observed that p16^INK4A^ blocks E2F function and thus inhibits α-klotho promoter activity to accelerated senescence [61]. The p16^INK4a^ prevents inactivation of retinoblastoma (Rb) phosphorylation by inhibiting cyclin D-dependent kinases. Then Rb represses E2F transcription factors expression by recruiting histone deacetylases to its promoter. The activated retinoblastoma (Rb) pathway simultaneously promotes formation of senescence-associated heterochromatic foci (SAHF), similarly refining the senescence-promoting mechanism of p16^INK4a^ [62].

It is controversial whether there are side effects of p16^INK4a^ -positive cells ablation. Removal of p16^INK4a^ positive cells can lead to the side effect of fibrosis in the liver and perivascular tissue, which in turn reduces life expectancy [63]. Partial reprogramming of senescent cells is therefore one of the possible solutions to this problem.

#### 3.2.2. Senescence-Associated Secretory Phenotype (SASP)

On the whole SASP is detrimental. For example, less than 1% of senescent preadipocytes can cause extensive physical dysfunction in young mice [13]. The killing of adjacent normal cells by SASP affects organ function [64], causing secondary senescence and increasing the accumulation of senescent cells that cause a variety of chronic inflammation/diseases [37] (senescent cells themselves are stalled in replication, so their primary cause of increased senescence is secondary senescence [64]). The anti-apoptotic capacity of senescent cells also increases the accumulation of senescent cells, and this property also protects these cells from SASP (creating a vicious circle) [65]. Targeted reprogramming of these cells may kill cells by breaking the anti-apoptotic capacity of senescent cells (it has been demonstrated in vivo in acute myeloid leukaemia cells, where short-term activation of OSKM expression induces apoptosis in leukaemic cells with little effect on normal haematopoietic stem and progenitor cells [66]). However, another possibility is to retain the beneficial components while eliminating the harmful ones (senescent cells are heterogeneous, and one subpopulation is beneficial for reprogramming and regeneration [14]). The validation of this hypothesis is one of the valuable directions for future research, so this section will comment on the beneficial potential of SASP.

Cells with p16^INK4a^ promoter activation were monitored in vitro and in vivo to accumulate senescence and inflammation. They showed senescence features such as reduced cell proliferation and activation of senescence-associated β-galactosidase (SA-β-gal). Additionally, they augmented the expression of genes related to the SASP [67]. The biological conditions associated with ageing, p16^Ink4a^, create a relaxed tissue environment by producing the cytokine interleukin 6, which supports reprogramming of OSKM in vivo [6].

Skeletal muscle and (white) adipose tissue are two tissues that develop phenotypes associated with early senescence in response to BubR1 dysfunction, and they are high in p16^INK4A^ and p19^Arf^ [58]. p16^INK4A^ inactivation in BubR1-deficient mice attenuated cellular senescence and premature senescence in these tissues. In contrast, p19^Arf^ inactivation exacerbated senescence and senescence in BubR1 mutant mice. Thus, BubR1 functional incompetence triggers Cdkn2a locus activation in some mouse tissues [58]. p16^INK4A^/p19^Arf^-free tissue attenuates cellular senescence and reduces IL6 production and reprogramming efficiency. Tissues without p53, on the other hand, are extensively damaged and senescent, create high levels of IL6, and are efficiently reprogrammed. Thus p16^INK4A^, but not p19^Arf^, is required for OSKM-induced senescence and paracrine stimulation [68].

Specific removal of senescent cells, instead, reduces reprogramming effectiveness, and the outcome of senescence on reprogramming is mediated in part by interleukin-6 (IL-6) [7]. Thus, SASP promotes reprogramming, but reprogramming decreases SASP and thus can create a weak negative feedback regulation. On the plus side, it may prevent loss of organ function and teratomas caused by excessive reprogramming. However, it may also result in less efficient reprogramming. In addition to selecting safe tissues for reprogramming, one should also consider the extensive linkage of the selected tissue to the whole body and the simultaneous regulation of factors with synergistic effects in anti-ageing.

In addition to its contribution to reprogramming, SASP, as a major dynamic component of the senescence microenvironment, also assumes a role in regulating the cellular senescence state. Senescent cells can transfer proteins directly to neighbouring cells, and this cellular communication enhances the immune surveillance of cell senescence by natural killer (NK) cells [52]. A direct attempt to exploit this mechanism is to liberate NK cells from inhibition to target senescent cells for killing. This strategy shares a feature with two other strategies (i.e., first, targeting senescent cells for killing by means of chimeric antigen receptor T (CAR T) cells and NK cells [69], and second, 2-BCL-2 inhibition to induce apoptosis to kill senescent cells [70]).

Senescence transmission has been found to be transmitted via soluble factors and extracellular vesicles (sEVs) that makeup SASP [71]. In addition to SASP, Ras (rat sarcoma viral oncogene)-induced senescence through a juxtacrine NOTCH1 (Notch Receptor 1)–JAG1 (Jagged1) pathway contributes to senescence in adjacent cells, defined as “secondary senescence” [72,73]. This shows the intercellular transmission of the senescence state (NOTCH1/JAG1) and the potential of SASP secreted by senescent cells as a key node in anti-ageing strategies. After reprogramming deeply aged cells with OSKM, the senescent microenvironment is transformed into a rejuvenating microenvironment by altering SASP; multiple substances in the rejuvenating microenvironment metabolically remodel moderately aged cells (improved mitochondrial function) and then remodel mitochondrial nucleosome interactions such as ROS-DDR. The rejuvenation microenvironment is characterized by a wide range of substances that reshape the metabolism of moderately senescent cells (improved mitochondrial function). SASP is thus an essential part of the microenvironmental remodelling and intercellular communication; another part of SASP’s role is to link the endocrine (e.g., eNAMPT) and immune systems (e.g., glutamine) in this strategy to amplify the effects and scope of anti-ageing, which is described below (Figure 2).

#### 3.2.3. DNA Methylation Level (Epigenetic Clock)

Epigenetics, characterized by acetylation and methylation (especially methylation of histone and the cytosines of CpG dinucleotides [35,74]), plays an essential role in cellular ageing. Thus, the “epigenetic clock (using the key age-related CpGs in a weighted linear model to predict chronological age)” might be indicative of biological age [37]. In addition, multiple studies have shown that epigenetic rejuvenation is possible through partial reprogramming, as reflected by age-deceleration in epigenetic clocks [37]. Therefore, epigenetic remodelling might be one of the most important ways to achieve a synergistic reversal of ageing. A genome-wide knockdown screen of human embryonic stem cells carrying a premature ageing mutation (CRISPR-Cas9-based) revealed that inactivation of the histone acetyltransferase KAT7 could inhibit p15^INK4b^ transcription by reducing acetylation of histone H3 lysine 14 (H3K14) and is anti-ageing [75]. Reprogramming resets telomeres in supercentenarian cells, implying its massive role in cell rejuvenation [76]. Even with extensive epigenetic defects, reprogramming can still reset the epigenetic pattern to a revitalized pluripotent state [77].

#### 3.2.4. Telomeres

In addition to remodelling the epigenetic landscape, partial reprogramming also prolongs telomeres in senescent cells [31]. Telomeres are repetitive nucleotide sequences located at the ends of chromosomes, are directly linked to cellular senescence, and are regulated by telomerase. Telomerase is a ribonucleoprotein complex that in humans consists of an enzyme, telomerase reverse transcriptase (TERT), plus a non-coding human telomerase RNA (hTR). The latter acts as a template for the prolonging of telomer length at the ends of chromosomes [78].

Tert overexpression significantly delayed ageing in mice by slowing telomere wear and preserving stem cell proliferative potential, but this required an increase in tumour suppression to counteract the pro-tumorigenic effects telomerase [40]. Abnormal telomere function inhibits PGC-1α and its downstream gene network via the p53-PGC pathway, thereby affecting cellular metabolism, causing organ dysfunction and leading to ageing [41]. Transient activation of telomerase restores neurogenesis in the subventricular zone and improves odour detection, suggesting that telomere lengthening reverses neural ageing and enhances its regenerative capacity, broadly improving organ function [79]. Therefore, tissue-specific transient telomere activation appears to be beneficial. For example, studies of telomerase gene therapy in mice by expressing pancreatic TERT with a broadly targeted adeno-associated virus (AAV) have also achieved beneficial effects, including increased pancreatic lifespan with reduced insulin sensitivity, osteoporosis, neuromuscular coordination, and molecular markers of ageing but no additional cancers occurred, which may suggest that the known oncogenic activity of telomerase is reduced when expressed in adult or aged organisms using an AAV vector [80].

In vitro assays with fibroblasts obtained from Tert knockout mice showed that mTert^−/−^ cells are more susceptible to senescence and malignancy than mTert^+/+^ cells. Telomerase reverse transcriptase (TERT) expression is upregulated by mTert^+/+^ cells prior to senescence. In addition, knockdown or downregulation of TERT by CRISPR/Cas9 or shRNA reproduced the mTert^−/−^ phenotype, while overexpression of TERT in mTert^−/−^ cells was rescued [81]. In summary, whether transient induction of telomerase expression is beneficial should also be investigated in different tissues in vivo, but the vast differences in the oncogenic capacity of human and mouse limit the potential application of telomerase. 

The activation of yes-associated protein 1 (YAP1), which upregulates the pro-inflammatory factor interleukin-18, can be rescued by mTert reactivation in mice with telomere dysfunction. In contrast, conventional SASP (IL-1, IL-6, IL8) did not show much change [82]. Thus, reprogramming strategies (which can regulate more inflammatory factors while lengthening telomeres) may have a synergistic effect on reducing the ageing-related chronic inflammation.

#### 3.2.5. Youthful Secretory Phenotype (YSP)

The youthful secretory phenotype is a newly proposed hypothesis. It aims to generalize the anti-ageing factors (including GDF11, GPLD1, clusterin, Klotho, NAD^+^, eNAMPT, GSTM2, exosomes, et al.) found in young blood and in the secretome of young cells [20,21,22,23,49,51,57]. These “young factors (existing in young blood as well as being secreted by a heterogeneous subgroup of ageing cells)” may dilute or inhibit “old factors” promoting ageing, thus playing a rejuvenating role [14,15].

Plasma proteome alteration can also interfere with senescence through intercellular and organ–organ communication [19]. Exposure of aged mice to young serum improved regeneration of senescent satellite cells (through Notch signalling activation), increased senescent hepatocytes’ proliferation, and restored the cEBP-α complex to youthful levels [83]. It is shown that blood input from young donors to elderly recipients improves the latter’s senescence-related phenotype. It is not unique that the soluble factors and extracellular vesicles (sEVs) that make up SASP can influence other cells’ senescence state and even transmit senescence by secretion [71]. Therefore, it is tempting to think that the “youthful secretory phenotype (YSP)” (secreted by young cells) could also convey youth across the whole body. For example, neonatal umbilical cord (UC)-derived mesenchymal stem cell extracellular vesicles (MSC-EV), which are rich in anti-ageing rejuvenation signals, rejuvenate senescent adult bone marrow-derived mesenchymal stem cells (AB-MSC) [84]. Exposure of neonatal umbilical cord-derived MSC extracellular vesicles (UC-EV) increased telomere length in AB-MSC with a significant improvement in SASP. It improved age-related degeneration of mouse bones and kidneys at the organ level.

After blood alteration, cardiac hypertrophy and cardiomyocyte size decreased significantly in old mice with concomitant molecular remodelling [85]. Growth and differentiation factor 11 (GDF11) could be one of the “rejuvenating” factors in young blood. Restoring circulating GDF11 levels reverses functional and genetic damage in aged muscle stem cells [86]. However, it remains controversial whether blood exchange therapy works by restoring GDF11 in aged mice to youthful levels [86,87]. For example, whether young blood improves synaptic plasticity and cognitive function through the activation of cyclic AMP response element-binding protein (Creb) in dentate gyrus neurons [88] or by enhancing neurogenesis in ageing mice via GDF11 [20].

In addition, the mechanism of young blood combating senescence remains controversial. The autophagic activity of aged livers can be restored by exposure to plasma from juvenile donors. Conversely, inhibition of autophagic activity eliminates the anti-ageing effect of plasmapheresis on the liver [89]. Exosomes from young serum significantly downregulated senescence-related genes (cyclin-dependent kinase inhibitor 2A, mechanistic target of rapamycin, and insulin-like growth factor 1 receptor) and upregulated telomerase related genes (e.g., Men1, Mre11a, Tep1, Terf2, Tert, and Tnks) in lung and liver by reversing mmu-miR-126b-5p levels of aged mice [90]. Thus, circulating anti-ageing factors are not unique and may work together through different pathways to exert anti-ageing effects. Exercise stimulates the liver to produce glycosylphosphatidylinositol-specific phospholipase D (GPLD1) [21]. It cannot cross the blood–brain barrier; instead, it improves age-related cognitive decline by reducing inflammation and increasing blood supply to the brain [21]. Thus, remote mediating mechanisms between organs can be both directly and indirectly anti-ageing. Not limited to organs such as the liver, kidney and brain, the cytokines MCP-1 and IL-6 (pro-inflammatory) were found to be reduced in visceral adipose tissue (VAT) of aged (18 months) mice by exposure to young plasma (from 3-month-old mice). Ageing adipose tissue-derived stromovascular fraction cells showed a decrease in the expression of the senescence markers (p16^Ink4a^ and p21^Waf1/Cip1^) [91]. Thus, amelioration of ageing-related hypofunction by providing a rejuvenating microenvironment (plasma proteome alteration) for senescent cells is widely applicable in a wide range of tissues [18].

In addition to blood-based evidence, this “youthful secretory phenotype (YSP)” (secreted by young cells) anti-ageing phenomenon is also widely observed in a variety of tissues (e.g., muscle, adipose, etc.). Cardiac stem cells are absent from the adult myocardium, but paracrine effects derived from young cardiomyocytes lengthen the telomeres not restricted to senescent cardiomyocytes. In aged rats treated with cardiac sphere-derived cells (CDCs) from young donors, circulating levels of the inflammatory cytokines interleukin-1β and interleukin-6 were reduced, along with elevated anti-inflammatory interleukin-10 levels, correlated with the observed improvements in exercise capacity, muscle reduction, hair regeneration, and renal function [92]. This research shows that young source tissue cells deliberately produce systemic benefits through systemic improvements rather than local improvements in single-organ ageing alone. Allogenic CDC intracoronary infusion in patients with heart attack increased left ventricle (LV) volume and N-terminal prob-type natriuretic peptide (NT-proBNP) compared with placebo but did not reduce scarring [93].

Further studies have shown that the ageing bone marrow can be reconstituted by tail vein transplantation of juvenile-derived antigen 1 positive (Sca-1+) bone marrow (BM) stem cells. It also promotes the rejuvenation of the heart by activating the c-x-c motif chemokine ligand 12 c-x-c chemokine receptor type 4 (Cxcl12/Cxcr4) pathway in cardiac endothelial cells [94]. CDC transplanted into the rat heart after myocardial infarction can derive exosomes into the bloodstream to promote myocardial repair via microRNAs associated with myocardial recovery [95].

**Figure 3:**
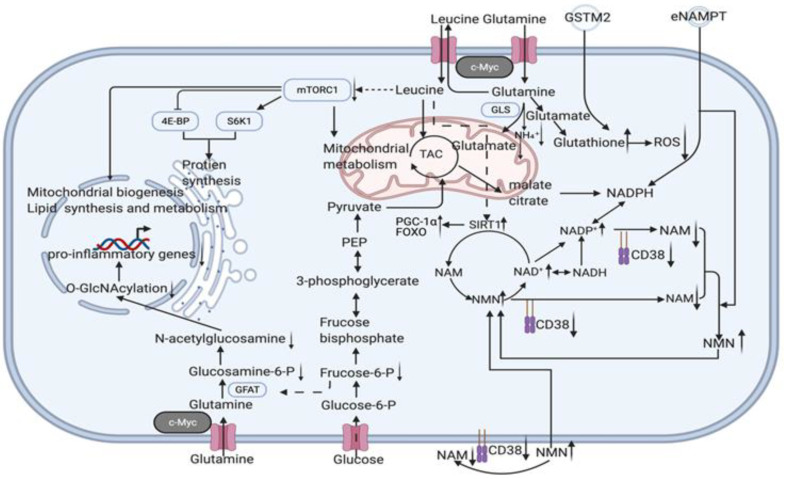
Potential intracellular mechanisms related to senescent cells specific reprogramming. Youthful secretory phenotype reverses the upregulation of cellular glutaminase 1 [56], so more leucine is transported into adipocytes with glutamine export. Leucine activation of mTORC1 and p70 ribosomal protein S6 kinase 1 (S6K1) promotes lipolysis and inhibits fatty acid synthesis [101]. However, its overall gains are obtained by increasing leptin and lipocalin secretion and/or synthesis in adipocytes, activating AMPK/SIRT1/PGC-1α signalling to regulate mitochondrial metabolism, and inhibiting the detrimental factors associated with mTORC1 activation to promote browning and fatty acid oxidation [102]. Therefore, in addition to amplifying the anti-ageing effect, the use of synergistic effects should also be considered to offset each other’s side effects to achieve an overall benefit to highlight the superiority of this strategy. For example, direct intake of leucine causes inhibition of Sestrin2, and thus activation of the rapamycin complex 1 (mTORC1) pathway, which shortens lifespan [103]. However, by improving adipose tissue function, rejuvenated adipose precursor cells produce more functional adipocytes, as we know that branched chain amino acids (BCAAs) promote adipose precursor cell differentiation, which in turn reduces acetyl coenzyme A (AcCoA) production from sugar and glutamine (thus increasing glutamine cycle levels and facilitating leucine entry into adipocytes) and increase branched chain amino acid (BCAA) catabolic fluxes [104]. BCAA catabolism, which reduced leucine levels in other tissues (thereby derepressing Sestrin2 inhibition and inactivating the rapamycin complex 1 (mTORC1) pathway). This leucine distribution (adipocyte enrichment) facilitates the activation of AMPK/SIRT1/PGC-1α signalling to regulate mitochondrial metabolism while suppressing the detrimental effects of mTORC1 activation in other tissues. Just as senescent adipocytes maintain survival by passing mitochondria to macrophages [54,105], increased glutamine catabolism in senescent cells to lower intracellular pH (glutamine-glutamate+NH₄⁺) is a way to save themselves [56], and the susceptibility of adipose to senescence leads to high amide consumption, which reminds us that macrophages secrete large amounts of SASP due to elevated glucolysis in a low glutamine environment [48]. Thus, increased glutamine levels inhibit glucolysis and thus improve inflammation, while GSTM2 in exosomes provides more GSH to maintain redox homeostasis [51]. In combination with the promotion of glycine adipocyte enrichment and the reduction in mTORC1 activation in other tissues, this is a synergistic anti-ageing effect. Furthermore, due to eNAMPT and cell rejuvenation, excess branched-chain amino acids are consumed via the tricarboxylic acid cycle, thereby retaining their role in promoting leptin secretion and Sirt1 activity while preventing excessive mTORC1 activation. CD38 expression increases with macrophage senescence, which can lead to a significant depletion of NAD^+^ [11,12], combined with a decrease in Sirt1 [43] function and eNAMPT [49,50] secretion triggered by adipocyte senescence and a decrease in the NAD^+^/NADH ratio [3]. This series of imbalances leads to mitochondrial dysfunction and an imbalance in redox status. The removal of these cells will delay senescence by reducing the above disorders, but the rejuvenation of these senescent cells may be achieved through the tricarboxylic acid cycle and the rearrangement of the disordered metabolic and transcriptional state through de novo synthesis and salvage production pathway (NAM–NMN–NAD^+^). The activation of Sirt1 has an anti-ageing effect via the PGC-1α and FOXO pathways [106,107,108]. (black arrow: direct stimulatory, round arrow: cycle, dotted arrow: tentative stimulatory, down faded arrow: decrease, up faded arrow: increase).

Nicotinamide adenine dinucleotide (NAD) is a metabolic cofactor that decreases with age and supplementation with its precursors, most commonly nicotinamide (NAM), is thought to be anti-ageing. Although NAM is a NAD^+^ precursor, NAM inhibits the NAM salvage pathway without producing a net elevation in the NAD metabolome. NAM treatment increases global protein acetylation, indicating that total sirtuins are inhibited. Combined with the reduction in nicotinamide phosphoribosyltransferase (NAMPT) levels it causes [96], the upper limit of the effect of NAD^+^ precursor supplementation alone may not be sufficient to meet anti-ageing requirements (enzymatic reaction balance may have an inhibitory effect on enzymes and enzyme depletion and inactivation due to ageing). Therefore, the reduction in NAD depletion caused by heavy braiding, enzyme renewal, and mitochondrial rejuvenation may be more advantageous. The NAD^+^-dependent deacetylase (SIRT1) in mammalian adipocytes deacetylates intracellular nicotinamide phosphoribosyltransferase (iNAMPT) to facilitate secretion to form extracellular nicotinamide phosphoribosyltransferase (eNAMPT) and enhance eNAMPT activity. eNAMPT in adipose tissue enhances hypothalamic NAD^+^/SIRT1 signalling and physical activity [57]. On the other hand, the hypothalamus is thought to be the superior control centre for ageing in mammals. By influencing endocrine regulatory centres, eNAMPT links adipose reprogramming anti-ageing strategies to endocrine regulation. The levels of eNAMPT in extracellular vesicles (EVs) decreased significantly with age and increased the secretion of eNAMPT-containing EVs from adipocytes; it significantly improved the behavioural phenotype of ageing and prolonged lifespan in mice [49]. In addition to the direct effects on cellular energy metabolism and redox, we should also consider the indirect effects caused by eNAMPT, such as acetylation, which is widely involved in a variety of metabolic and chromatin regulations (histone modifications) and thus extensively affects mitochondrial metabolic remodelling and epigenetic remodelling in reprogramming strategies. Some easily overlooked mechanisms of gene damage also need to be investigated in an integrated manner to complete the network of crucial factors for reversal of ageing; e.g., in addition to ROS-dependent DDR, DNA-protein cross-linking (DPC) is also one of the triggers of nuclear senescence. Gene damage induces ATM/ATR activation, which activates the deubiquitinating enzyme VCPIP1/VCIP135 by phosphorylation, and VCPIP1 deubiquitinates SPRTN(a DNA-dependent metalloproteinase) to create the conditions for its subsequent acetylation, which eventually localizes SPRTN to chromatin damage sites to cleave DPCs proteins and protect genomic stability from senescence [97]. Thus eNAMPT and the response induced by changes in NAD^+^/NADH ratios (and even changes in sirtuin1,3) are by no means limited to the previously described crosstalk mechanisms between ROS-induced DNA damage response (DDR) and mitochondrial dysfunction-associated senescence (MiDAS) [3,4] but are instead more broad. Reduced glutamine levels in lipid cells reduce uridine diphosphate N-acetylglucosamine (UDP-GlcNAc) levels via glycolysis, reducing O-linked β-N-acetylglucosamine (O-GlcNAc). This ultimately leads to chronic inflammation development by promoting a pro-inflammatory transcriptional response through O-GlcNAcy of chromatin-binding proteins near inflammatory genes [48] (Figure 3).

Extracellular vesicles (EVs) secreted by young donor fibroblasts are rich in GST-active GSTM2, increasing the level of GSH in mice and humans and reducing ROS accumulation and lipid oxidation in aged mice and humans. This modulates brown adipose tissue (BAT) and kidney and lung senescence markers and influences the SASP in serum, thereby systematically improving the cellular and physiological characteristics of ageing [51]. Therefore the potential of sEVs in young individuals to ameliorate ageing-associated cell damage was investigated [51]. The secretion of sEVs by old individual cells (“old cells”) can induce paracrine senescence characteristics to the proliferating cells of young individuals (“young cells”) to facilitate their reprogramming [7]. Thus, the above two relationships can be partially reprogrammed to create a self-regulating virtuous cycle in the body. White adipose tissue infiltrated by CD38^+^ M1 macrophages in senescent animals leads to a decrease in the levels of NAD^+^ and its precursor, NMN. Knockdown of the CD38 gene prevented a decline in NAD^+^. In vitro co-culture of senescent cells and macrophages resulted in elevated CD38 expression and activity. Inhibition of SASP reduced CD38 in vivo, thereby reversing the decline in NAD^+^ levels. It was shown that senescence-induced infiltration of CD38^+^ macrophages in white adipose tissue overexpressing CD38 leads to an overall decrease in NAD^+^. Small amounts of CD38 present in cells are sufficient to regulate their NAD^+^ levels, but CD38 can also reduce NAD^+^ levels by blocking their use of extracellular NMN. Exposure to CD38 antibody reversed the decline in NAD^+^ levels in white adipose tissue of aged mice, and the effect was superimposed on NMN supplementation [11,12]. Thus, the introduction of adipose reprogramming can also broadly regulate multiple organs throughout the body (vital metabolic organs such as the hypothalamus, liver, and pancreas) by modulating CD38^+^ macrophages and circulating eNMPT and GST in both immune and cellular metabolic pathways.

The mmu-miR-126b-5p in young serum-derived exosomes downregulates senescence-related genes in (p16^Ink4A^, MTOR, and IGF1R) in lung and liver tissues of aged mice and upregulates telomerase-related genes (e.g., Men1, Mre11a, Tep1, Terf2, Tert, and Tnks) in the liver to show anti-ageing potential [90]. An earlier study showed that transfection of miR-21a-5p, miR-103-3p, and miR-30c-5p decreased Mre11a, p16^INK4a^, and Mtor in the liver of aged mice [98]. Mmu-miR-291a-3p in mouse ESC exosomes decreased the activity of β-galactosidase, improved proliferation, and decreased expression and translation of TGF-β receptors 2, p53, and p21 in senescent cells. The human homologs of mma-miR-291a-3p, hsa-miR-371a-3p, and hsa-miR-520e, exercise comparable anti-ageing activity [99]. On the other hand, increased miR-29b-3p in exosomes released by BM-MSC in ageing mice, and consequently inhibition of sirtuin 1 (Sirt1), lead to increased senescence-associated insulin resistance [100].

## 4. Potential Candidates of Clinical Translation Scheme to Achieve Senescent Cells Specific Reprogramming

Partial reprogramming can be achieved by the transient expression of nuclear reprogramming factors transfected by mRNAs [36] or by AAV delivery [37]. The other methods used for complete reprogramming are also potent for applying partial reprogramming by drug control, for example, by injecting plasmids encoding OSKM by the single hydrodynamic tail vein (HTV) [109]. The effective cell permeability of recombinant transcription factors was established by fusing it with the minimal transduction domain of ZEBRA protein, triggering human fibroblasts’ regeneration and CD34+ cells without genetic interference and becoming one of the candidate approaches for a senescent cell-specific partial reprogramming strategy [110]. Even reprogramming senescent cells with a low-dose OKSM delivery vector of pseudo typic adeno-associated virus (AAV) using AAV-DJ-coated shells is also an alternative approach [111]. However, since the amount of protein overexpressed by AAV is itself low, it does not indicate its long-term safety, and the same AAV-mediated reprogramming of OSK is more advantageous due to the removal of the oncogene c-Myc. It would also be interesting to investigate whether there is a synergistic anti-ageing effect of simultaneous overexpression of TERT and OSK (OSKM lengthens telomeres, the effect of OSK on telomere length is currently unknown [37]). It is also suggesting that specific induction of overexpression in pioneer senescent cells is a more favourable strategy than uncontrollable direct overexpression. Customized solutions and a safe organization’s choice are the directions that it will take to improve this strategy.

Pioneer transcription factors are defined as a class of transcription factors that can differentially bind DNA-binding domains (DBDs) to silence chromatin targets and initiate cell fate trajectory shifts [112]. In a reprogramming strategy, the TFs OSK act as pioneer transcription factors in reprogramming to target the LIN28B motif, a key site for reprogramming and pluripotency on nucleosomes, and binding to Myc, which cannot target nucleosomes alone, to direct its targeting to the condensed E-box on nucleosomes and together regulate cell fate [112]. Some studies have shown that OSK combinations alone still have an anti-ageing effect [37,113], but Myc, as an efficiency enhancer, greatly enhances reprogramming efficiency [114]. There is a critical role of Myc for glutamate metabolism. Overexpression of c-Myc increases glutamine levels in lipocytes, thereby decreasing glycolysis, and the glycolytic pathway produces less uridine diphosphate N-acetylamino glucose (UDP-GlcNAc), further decreasing nuclear O-linked β-N-acetylamino glucose (O-GlcNAc)). The reduced level of O-GlcNAc in the nucleus restricts the O-GlcNAcylation of inflammatory gene-associated chromatin-binding proteins, thereby inhibiting pro-inflammatory transcription [48]. This will lead to attenuated chronic inflammation caused by enriched immune cells in the vicinity of white adipocytes and through macrophages in tissues, attenuating NAD^+^ depletion in vivo [11,12]. This cascade breaks the ROS-induced DNA damage response by increasing the NAD^+^/NADH ratio (DDR) and mitochondrial dysfunction-associated senescence (MiDAS) crosstalk [3,4] while reshaping the rejuvenation pattern at the epigenetic, metabolic, and redox levels and mitochondrial metabolism. Mitochondria regulation also goes beyond energy metabolism and redox; as described above, the function of mitochondrial DNA should also be considered. For example, Humanin, a peptide encoded by a short open reading frame within the mitochondrial genome, can extend the lifespan through the daf-16/Foxo pathway [115]. Therefore, comparing the efficiency and side effects of OSKM and OSK schemes in anti-ageing is also a way to resolve the controversy (which is superior in anti-ageing).

## 5. Conclusions

Senescence-specific phenotypes are manifested by increased expression of senescence-associated genes (senescence transcriptional program, epigenetic alterations, telomerase shortening, senescence-associated chronic inflammatory state) and altered metabolic state (altered mitochondrial function, redox imbalance, disturbances in the metabolism of key molecules of senescence, such as NAD^+^), while cell cycle (cell cycle withdrawal) and protein synthesis (ribosomal ageing phenotype, senescence-associated endocrine phenotype) also appear to be characteristically altered. Of these, the senescence-associated endocrine phenotype (which encompasses the indirect cellular communication of all senescent cells with immune cells, young cells, and organ-specific functional cells) is an essential component of the senescence microenvironment. The multiple cytokines, enzymes, and extracellular vesicles (EVs) [71] that make up the SASP can interact with young cells through the senescence microenvironment, a balance that generally promotes senescence. Still, the rejuvenating microenvironment of immature cells can also improve the metabolic state of senescent cells at the tissue level and thus break the senescence signature within senescent cells through the remodelling of protein synthesis and gene expression. It is possible that the vicious cycle of senescence within senescent cells can be broken through the remodelling of protein synthesis and gene expression patterns. For example, blood-derived factors and extracellular vesicles of young origin have been shown to rejuvenate senescent mice [71,83], implying that breaking the dominance of the senescent microenvironment in the senescent organism and changing this balance to one dominated by the rejuvenating microenvironment has the opportunity to reprogram the metabolism of senescent cells and thus break the characteristic cycle of senescence within senescent cells. 

Additionally, the senescent microenvironment itself promotes cellular reprogramming, which may be an opportunity left by evolution to combat senescence with controlled reprogramming of local tissues (based on the OSKM Yamanaka factor, which essentially creates a persistent young environment in a controlled manner), in turn, radically improving the overall senescence homeostasis of senescent cells through metabolic reprogramming and epigenetic remodelling [51], and this deadlock-breaking anti-ageing strategy is autonomously regulated by the ageing microenvironment, depending on the degree of senescence (the more the microenvironment is inclined to senescence, the easier the local reprogramming, metabolic reprogramming, and epigenetic remodelling). 

In summary, the phenomena we expect to see in future research and clinical translation are as follows: As rejuvenation becomes more pronounced, local reprogramming loses the promotion from SASP and combines with a controlled induction system to avoid tumours and loss of cellular function. It must be stressed that this strategy must be implemented in non-fatal tissues (such as adipose, fascia rather than myocardium, and nerves).
